# The N-Acetylglucosamine Kinase from *Yarrowia lipolytica* Is a Moonlighting Protein

**DOI:** 10.3390/ijms222313109

**Published:** 2021-12-03

**Authors:** Carmen-Lisset Flores, Joaquín Ariño, Carlos Gancedo

**Affiliations:** 1 Instituto de Investigaciones Biomédicas “Alberto Sols” CSIC-UAM, 28029 Madrid, Spain; carl.gancedo@gmail.com; 2 Institut de Biotecnologia i Biomedicina & Departament de Bioquímica i Biologia Molecular, Universitat Autònoma de Barcelona, 08193 Cerdanyola del Vallès, Spain

**Keywords:** moonlighting, N-acetyglucosamine kinase, *Yarrowia*, yeast, N-acetyglucosamine transporter

## Abstract

In *Yarrowia lipolytica*, expression of the genes encoding the enzymes of the N-acetylglucosamine (NAGA) utilization pathway (*NAG* genes) becomes independent of the presence of NAGA in a *Ylnag5* mutant lacking NAGA kinase. We addressed the question of whether the altered transcription was due to a lack of kinase activity or to a moonlighting role of this protein. Glucosamine-6-phosphate deaminase (Nag1) activity was measured as a reporter of *NAG* genes expression. The *NGT1* gene encoding the NAGA transporter was deleted, creating a *Ylnag5 ngt1* strain. In glucose cultures of this strain, Nag1 activity was similar to that of the *Ylnag5* strain, ruling out the possibility that NAGA derived from cell wall turnover could trigger the derepression. Heterologous NAGA kinases were expressed in a *Ylnag5* strain. Among them, the protein from *Arabidopsis thaliana* did not restore kinase activity but lowered Nag1 activity 4-fold with respect to a control. Expression in the *Ylnag5* strain of YlNag5 variants F320S or D214V with low kinase activity caused a repression similar to that of the wild-type protein. Together, these results indicate that YlNag5 behaves as a moonlighting protein. An RNA-seq analysis revealed that the *Ylnag5* mutation had a limited transcriptomic effect besides derepression of the *NAG* genes.

## 1. Introduction

The aminosugar N-acetylgucosamine (NAGA) is a constituent of a variety of important biological molecules such as chitin, hialuronic acid or the bacterial peptidoglycan. Chitin, a polymer composed of repeating NAGA units, is the most abundant polymer in nature after cellulose, with a biosynthetic output in the range of 10^11^–10^14^ tons/year [1]. It could be expected, therefore, that organisms able to use NAGA as carbon, or even nitrogen source, would have been selected in the course of evolution and, in fact, a number of bacteria and fungi are able to grow in NAGA as sole carbon source [2,3,4,5,6,7]. Curiously, among the more than 1500 yeast species identified, only a few are able to grow in NAGA as carbon source [8]. One of these is *Yarrowia lipolytica*, a non-pathogenic, dimorphic yeast, distantly related to other ascomycetous yeasts [9] that has been used in the study of dimorphism, peroxisome biogenesis and the mitochondrial complex I (for a review see [10]). Several characteristics of *Y. lipolytica*, such as the regulatory properties of some of its glycolytic enzymes [11,12] or the transcriptional control of genes subject to catabolite repression [13,14], are considerably different from those of the model yeast *Saccharomyces cerevisiae*. The status of *Y. lipolytica* as a GRAS organism (Generally Recognized As Safe) [15] and other outstanding properties of this yeast, such as its high capacity to secrete proteins or to use unusual growth substrates, have fuelled the interest of the industry to use it in various processes [16,17] and to develop new tools to expand its capabilities [18].

**Figure 1:**
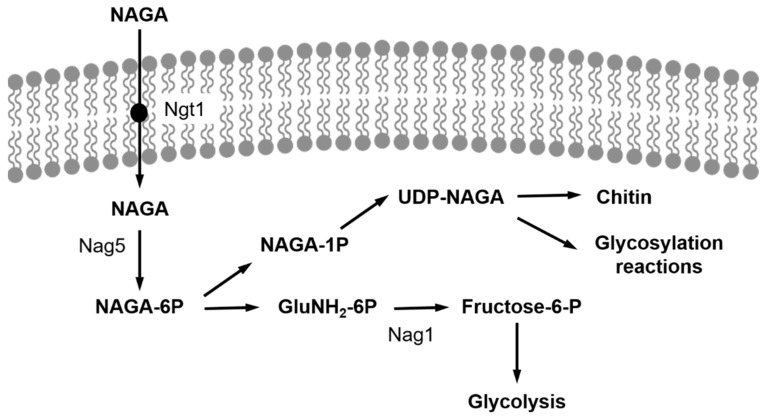
Pathway of NAGA utilization in *Y. lipolytica*. After its transport by Ngt1, NAGA is phosphorylated by NAGA kinase, Nag5, and channelled to the glycolytic pathway. NAGA-6-P is also an initial metabolite for chitin synthesis and glycosylation reactions. Nag1, glucosamine-6-P deaminase.

The pathway of NAGA utilization by *Y*. *lipolytica* has been characterized [19] and is similar to that found in other organisms. It requires a transport step followed by an intracellular pathway that comprises the phosphorylation of the amino sugar by a NAGA kinase, encoded by the gene *YlNAG5* [19], a deacetylation step and a deamination-isomerization yielding fructose-6-phosphate that may be incorporated into the glycolytic pathway (Figure 1). The transcription of the genes encoding the enzymes of the pathway (*NAG* genes) is regulated by the presence of NAGA in the culture medium: in a wild-type strain, those genes are expressed at a very low level during growth in glucose, while their expression increases 20- to 40-fold in cultures grown with NAGA as carbon source [19]. However, a deletion of the gene *YlNAG5*, encoding the NAGA kinase, abolishes this regulation and renders the transcription of those genes independent of the presence of NAGA in the medium [19].

A possible explanation for this observation is that Nag5 is a moonlighting protein with two functions: one as a metabolic kinase, the other as a regulatory protein implicated in the control of the expression of the genes of the NAGA catabolic pathway. Moonlighting proteins are proteins that perform disparate functions and that are not the result of gene fusions, or RNA splice variants [20,21]. An alternative explanation would be that the transcriptional deregulation of the *NAG* genes in the mutant is triggered by an intracellular NAGA accumulation derived from chitin turnover. Our objective in this work was to distinguish between these two possibilities. To this end, we studied the regulation of the expression of the *NAG* genes in a double mutant *Ylnag5 ngt1* that cannot transport NAGA and in a *Ylnag5* mutant expressing heterologous NAGA kinases or mutated versions of *Yl*Nag5. Additionally, we performed RNA-seq experiments to monitor the effects of the *Ylnag5* mutation on the expression of genes not related to the NAGA pathway. Our results are consistent with the idea of NAGA kinase being a moonlighting protein with two roles, one as metabolic enzyme and another as regulatory protein in the transcription of some genes.

## 2. Results

### 2.1. Enzymatic Activity of Glucosamine-6-P Deaminase as Reporter for the Expression of the NAG Genes

The expression of the *NAG* genes is dependent on the carbon source in the culture medium, being only elevated in the presence of NAGA. To ascertain the expression state of the *NAG* genes, we measured the activity of glucosamine-6-phosphate deaminase encoded by the gene *YlNAG1* (*YALI0C01419g*). Two premises should hold true to accept this assay as reporter of the expression of the *NAG* genes: first, the levels of *YlNAG1* mRNA should vary in parallel with the mRNA levels of the other genes of the pathway; second, the enzymatic activity of YlNag1 must follow the same pattern than its encoding mRNA both in the wild type and in the *Ylnag5* mutant under different culture conditions. The first premise was fulfilled, as measurements of RNA expression by qPCR showed that the *YlNAG1* gene was repressed during growth in glucose and induced ca. 27 times during growth in NAGA [19]. To test the second requirement, the Nag1 activity was measured in cultures of a wild-type strain and of a *Ylnag5* mutant strain grown in different carbon sources. For the wild type grown in NAGA, an activity of 227 ± 30 mU/mg protein was found *vs* < 1 mU/mg protein in a glucose grown culture. In the case of the mutant, unable to grow in NAGA, the activity in a glucose-grown culture was 250 ± 26 mU/mg protein in the same range as that of the wild type grown in NAGA. This result shows that the enzymatic activity parallels the behaviour of the mRNA [19] and validates the use of this enzyme as reporter for the derepression of the genes of the NAGA catabolic pathway.

### 2.2. Disruption of the Gene Encoding the NAGA Transporter Does Not Affect the Deregulated Expression of the NAG Genes in a Ylnag5 Mutant

**Figure 2:**
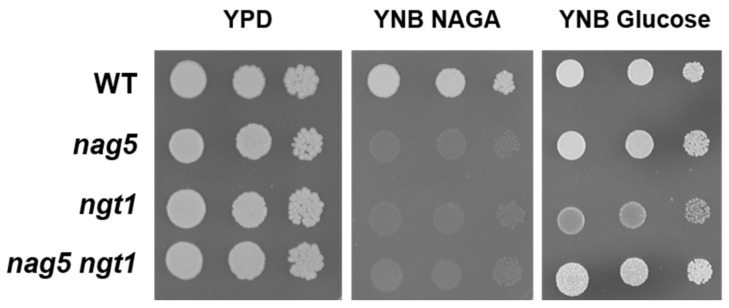
Growth behaviour of *Y. lipolytica* strains bearing disruptions in *YlNAG5* and *YlNGT1*. Seven microlitres of suspensions of about 10^6^ cells/mL and successive 10-fold dilutions of each strain were plated. They were grown in the indicated media at 30 °C. Pictures were taken after three days of incubation.

**Figure 3:**
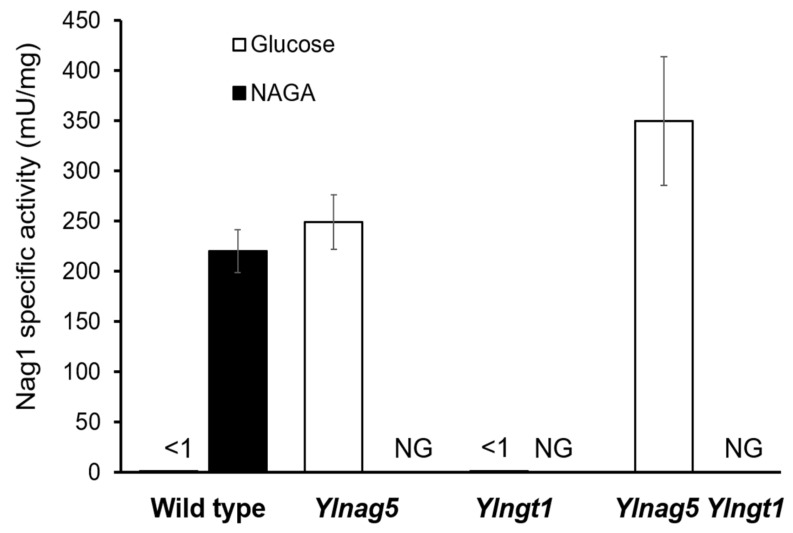
Specific activity of Nag1 in strains carrying disrupted versions of *YlNAG5* and *YlNGT1*. The indicated strains were grown in glucose or NAGA, and Nag1 activity was assayed as described in Material and Methods. The values are means with standard deviations from three independent cultures. The activity was <1 mU/mg protein in the cases in which no column bar is visible. NG, no growth.

A hypothesis to explain the altered expression of the *NAG* genes in a *Ylnag5* mutant growing in glucose could be that it is due to an intracellular accumulation of NAGA. A possible source of this amino sugar could be the hydrolysis of chitin occurring during cell wall remodelling, a process that occurs extracellularly. The uptake of NAGA requires a transporter, Ngt1, encoded putatively by the gene *YALI0D09801g* [19]. The mechanism of this transport is unknown, but if it were active, NAGA could accumulate in a *Ylnag5* mutant. Deletion of the *YlNGT1* gene in a wild-type strain abolished growth in NAGA (Figure 2), thus confirming its tentative assignation [19] and indicating that there is no other relevant transporter for this amino sugar in *Y. lipolytica*. A double *Ylngt1 nag5* mutant was generated, as described in Material and Methods, and the activity of Nag1 was measured in this strain. As shown in Figure 3, this activity was similar to that measured in the single *Ylnag5* mutant grown in glucose. This result indicates that the altered derepression of the *NAG* genes in a *Ylnag5* mutant in glucose cultures is not due to an internal NAGA accumulation.

### 2.3. Assays to Evaluate the Enzymatic and Regulatory Activities of YlNag5

**Figure 4:**
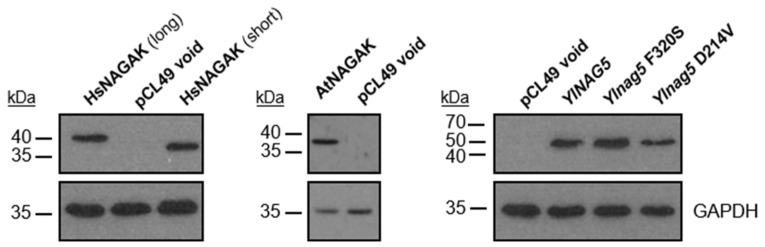
Immunoblot analysis of the expression of heterologous NAGA kinases and of mutated forms of *Y. lipolytica* NAGA kinase. Captions above each lane indicate the protein expressed. Glyceraldehyde 3-P dehydrogenase (GAPDH) is shown as control protein for loading and transfer efficiency. Treatment of samples, blot procedure and antibodies used in each case are described in Section 4.5 of Material and Methods.

To determine whether the metabolic activity of the NAGA kinase could be separated from its effect on the expression of the *NAG* genes, we examined the consequences of expressing in a *Ylnag5* mutant heterologous NAGA kinases or variants of *YlNAG5* with randomly generated mutations. The heterologous proteins chosen were the NAGA kinase from *Arabidopsis thaliana* and that of human origin, both of which have been biochemically characterized [22,23,24]. In addition, a protein from *Magnaporthe oryzae* (G4NB55) annotated as a hexokinase-like enzyme in the databases but predicted to be a NAGA kinase by a phylogenetic analysis [19] was used. Table 1 shows the results of the expression of heterologous proteins in a *Ylnag5* mutant. The strain transformed with the void plasmid maintained the features observed in the original mutant strain: no kinase activity and activity of Nag1 (representative of the *NAG* genes). The protein from *A. thaliana* was expressed as shown by Western blot (Figure 4), but the transformed strain did not grow in NAGA and no NAGA kinase activity was detected in extracts. Nevertheless, measurements of Nag1 activity in this strain showed that it was 4 times lower than that measured in the strain with a void plasmid (Table 1). Thus, the strain expressing the AtNAGA kinase recovered an important level of repression of the *NAG* genes, indicating that the kinase activity and its effect on the expression of the *NAG* genes could be separated. Expression of the putative kinase of *M. oryzae* (G4NB55) in the *Ylnag5* mutant allowed growth in NAGA and completely repressed expression of the *NAG* genes in a glucose medium. These results identify this protein as a *bona fide* NAGA kinase, although it is annotated as a hexokinase-like enzyme in the databases.

Regarding the human NAGA kinase, we noticed that in the manually curated annotation Havana (https://www.ensembl.org/info/genome/genebuild/manual_havana.html, accessed on 11 September 2013) of the human genome from the Vertebrate Genome Annotation, there is a transcript that encodes a protein 46 amino acids longer than the published sequence (Uniprot Acc. # Q9UJ70-2). Both, the “short” and “long” sequences, adapted to the codon usage of *Y. lipolytica*, were expressed in the *Ylnag5* mutant (Figure 4) and allowed its growth in NAGA and completely repressed expression of the *NAG* genes in a glucose medium.

**Figure 5:**
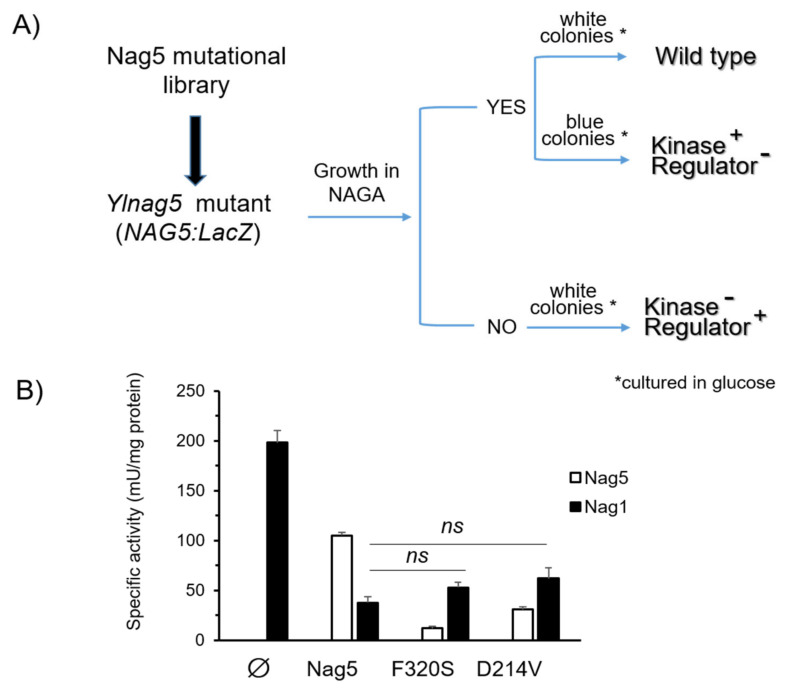
(**A**) Flow chart of the procedure to isolate mutant forms of YlNag5 fulfilling different functions of the protein. An *Ylnag5* mutant strain bearing a fusion of the *YlNAG5* promoter to *lacZ* was transformed with a library of randomly mutated *YlNAG5*; transformants were selected on glucose medium (denoted by an asterisk) and screened as shown in the figure. (**B**) Activity of NAGA kinase (Nag5) and NAGA-6-P deaminase (Nag1) in the indicated strains grown in glucose. Measurements are mean values of three independent cultures with their standard deviations. The differences in Nag5 activity between the mutated variants and the native Nag5 were highly significant (*p* < 5 × 10^−5^), while those of Nag1 activity were not significant (ns) as determined by 2-tailed Student’s test. ∅, denotes the void plasmid.

To look for mutant forms of YlNag5 able to fulfil only one of the two functions ascribed to the protein, we proceeded as follows. We constructed a strain with a deleted *YlNAG5* gene in which a fusion of the *YlNAG5* promoter to *lacZ* was inserted at the genomic *leu2* locus (see Material and Methods). This strain was unable to grow in NAGA and produced blue colonies in a glucose X-Gal plate. It was transformed with a randomly mutagenized library of *YlNAG5* (see Material and Methods) and transformants were grown on glucose and then replica-plated to NAGA (see flow chart in Figure 5A). Those growing on NAGA could yield either white or blue colonies on glucose X-Gal plates. The white ones will have a NAGA kinase with properties similar to those of the wild type; nine colonies of this type were isolated. The blue ones could have conserved the enzymatic activity but lost the regulatory one; 29 colonies of this type were isolated. The non-growers in NAGA will have lost the kinase activity but those producing white colonies on glucose X-Gal plates could have conserved the regulatory activity; no colonies of this type were found. We focused our attention on the transformants growing in NAGA and being blue in glucose. The corresponding plasmids were extracted and sequenced. All of them presented more than one mutation (Supplementary Table S1). In order to pinpoint single mutations that could be responsible for the phenotype considered, we followed two approaches. One was to identify residues in the YlNag5 sequence that appeared mutated in different clones derived from the mutagenic PCR, e.g., the triplet that encoded amino acid F320 was altered in several clones, in some cases yielding serine and in others resulting in tyrosine. The other approach was to compare the mutated YlNag5 sequence with an alignment of hexokinases, glucokinases and NAGA kinases from different species [19] to localize mutations in positions generally conserved. Mutations F320S and D214V were identified and selected in this way. We constructed NAGA kinase versions bearing these single mutations and expressed them in the *Ylnag5* mutant. *Ylnag5* strains expressing the F320S or D214V variants of YlNag5 exhibited low or moderate kinase activity, in contrast with a strain expressing the wild-type *NAG5* gene (Figure 5B). However, the activity of Nag1 was similar in all these strains. We conclude that the Nag5 enzymatic activity does not parallel its regulatory capacity.

### 2.4. Transcriptional Changes Caused by the Ylnag5 Mutation

To search for genes whose expression could be affected by the *Ylnag5* mutation, we performed an RNA-seq analysis comparing the *Ylnag5* mutant with the wild type grown in glucose. From a total number of 1–1.5 × 10^6^ clean mapped reads, our study quantified the expression of 5734 genes from *Y. lipolytica*. Using a threshold of ≥2-fold change, ca. 1.0% showed an increase in expression and ca. 2.5% exhibited a decrease in expression.

This relatively small impact on the transcriptome profile indicates that the mutation of *YlNAG5* causes a rather narrow and specialized effect. In spite of the relatively poor functional annotation of the *Y. lipolytica* genome, a gene ontology enrichment analysis was performed with the group of genes that were overexpressed in the mutant with respect to the wild type. Among them, the NAGA metabolic process that comprises the genes of the NAGA catabolic pathway was identified (Table 2). This result confirms our previous finding showing the derepression of these genes in the *Ylnag5* mutant in the absence of NAGA [19].

Another gene whose levels were strongly increased was *YALI0F04466g* that encodes a protein with high similarity to Gig1 from *Candida albicans* [25] and to Htc1 from *S. cerevisiae*. Expression of gene *YALIF04169g* encoding a protein similar to *S. cerevisiae* glycogen phosphorylase was also increased. Among the strongly repressed genes, we identified genes encoding diverse putative plasma membrane transporters. Other biological processes resulted enriched among the genes overexpressed or repressed in the *Ylnag5* mutant compared with the wild type but with the data available we cannot establish a biological connection between their behaviour and NAGA metabolism (Table 2).

## 3. Discussion

In *Y. lipolytica*, the genes encoding the enzymes of the NAGA utilization pathway are derepressed in a *Ylnag5* mutant lacking NAGA kinase [19]. To determine the cause of this anomaly, we used different approaches. First, we considered the possibility that, in the absence of this enzyme, during growth in glucose, NAGA derived from cell wall turnover might accumulate within the yeast and induce the transcription of the *NAG* genes. Yeast chitinases are located extracellularly [26,27,28]; therefore, NAGA produced by them should be transported into the cell to elicit any effect. Disruption of the gene *YlNGT1* (*YALI0D09801g*) showed that it is the only NAGA transporter in *Y. lipolytica*, and thus it eliminates the possibility of NAGA accumulation in the yeast. Since the levels of Nag1, used as a reporter of the expression of the *NAG* genes, were similar in the double mutant *Ylngt1 nag5* and in a single *Ylnag5* mutant, NAGA accumulation does not account for the regulatory changes occurring in the *Ylnag5* mutant.

We then proceeded to examine the possible moonlighting role of the YlNAGA kinase, trying to separate its metabolic activity from the regulatory one. One approach was the use of heterologous NAGA kinases. The usefulness of heterologous proteins to ascertain the moonlighting role of a protein has been shown in the case of the pyruvate carboxylase of the methylotrophic yeast *Hansenula polymorpha*, in which expression of a heterologous pyruvate carboxylase in an *H. polymorpha pyc* mutant could complement the metabolic function but not the moonlighting one [29]. Expression of *A. thaliana* NAGA kinase in a *Ylnag5* mutant did not result in measurable in vitro kinase activity in glucose-grown yeast, but it caused an important repression of the *NAG* genes, indicating that the kinase activity and its effect on the expression of the *NAG* genes could be separated. The fact that the *A. thaliana* protein, although expressed, did not allow growth of the *Ylnag5* mutant in NAGA indicates that it also lacks activity in vivo. It is worth noting that this protein had been biochemically characterized as a NAGA kinase [22]. A possible explanation for this result could be that, in *Y. lipolytica*, the protein is partially misfolded and this alteration has a deep impact in the catalytic activity. Codon usage is rather different in *Y. lipolytica* and *A. thaliana*, with a stronger tendency to the use of GC-rich codons in the former organism. As a result, several codons frequently used in *A. thaliana* are very rarely employed in the fungus [30]. As an example of the importance of the codon usage for the folding of proteins, it might be mentioned that even a change in a codon that results in a silent mutation in a mammalian protein causes a problem in folding that results in an alteration in substrate specificity without altering the protein level [31]. In contrast the proteins from *M. oryzae* and *H. sapiens* showed activity in extracts, allowed growth in NAGA and caused total repression. Surprisingly, overexpression of the endogenous kinase did not restore completely repression of the *NAG* genes, a result confirmed in RNA-seq experiments (results not shown). It could be hypothesized that an excess of YlNag5 titrates another protein acting in the repression process and makes it less effective. The different behaviour of NAGA kinases from diverse origins when expressed in the *Ylnag5* mutant could be due to the fact that NAGA kinases, although performing the same biochemical reaction, are relatively distant in sequence [19].

Another approach was the use of mutated versions of the YlNag5 protein. Two single substitutions, F320S or D214V, produced low or moderate kinase activity and caused repression in the same range of that measured in a strain overexpressing the wild-type *NAG5* gene that presented an elevated kinase activity. Moreover, the repression observed with both variants was similar to that measured in the mutant expressing the *A. thaliana* kinase that did not show activity. We conclude therefore, that the Nag5 enzymatic activity does not parallel its regulatory capacity. Those results are consistent with the idea that YlNag5 behaves as a moonlighting protein.

The fact that in the blue/white mutant screen, no mutants completely devoid of kinase activity but retaining the regulatory function were found, was unanticipated. A possible explanation could be that the mutational library was not saturated and that it would have been necessary to screen a very large number of mutants to find this kind of mutants. Another possibility is that mutations that affect regions near the active site change the structure of the protein in such a way that it also affects its regulatory activity.

In *C. albicans*, the absence of NAGA kinase causes, during growth in glucose, an increase in the expression of the genes *NGT1* and *NAG1* encoding, respectively, the NAGA transporter and the NAGA-6P deaminase [32]. In addition, it has been found that homozygous *hxk1*/*hxk1* mutants of *C. albicans* (*HXK1* designates in this yeast the gene equivalent to *YlNAG5*) exhibited filamentous growth in media in which a wild type grew in yeast form [32]. These findings suggest that the protein is involved in processes unrelated to its metabolic function. In rat cell cultures, NAGA kinase was found to participate in dendritogenesis of hippocampal neurons, and protein versions lacking enzymatic activity were as effective as the wild type in this role [33]. This suggests that murine NAGA kinase behaves also as a moonlighting protein with various different roles. All of these results fit with our proposal of Nag5 being a multifunctional protein across evolution.

The RNA-seq experiments showed that most of the genes with increased expression levels in the *Ylnag5* mutant grown in glucose corresponded to those involved in NAGA catabolism. Another gene induced to high levels in the mutant was *YALI0F0446g* that encodes a protein of unknown function whose equivalent in *C. albicans*, Gig1, is induced by NAGA and is required for sensitivity to nikomycin, an inhibitor of chitin synthase [25]. It is noteworthy that no orthologs of *GIG1* have been found in yeast species that lack NAGA catabolic genes, except for the *YPL067C* (*HTC1*) gene of *S. cerevisiae* [25]. The protein Htc1 belongs to a family named HTC (for histidine triad with channel) [34]. It has been shown that a *S. cerevisiae htc1* null mutation increases the toxicity of the expression of a huntingtin fragment consisting of an expanded polyQ repeat [35], but the physiological role of the protein has not been further elucidated. The induction of *GIG1* by NAGA in *C. albicans* [25] and the increased expression of *YALI0F0446g* in the *Y. lipolytica Ylnag5* mutant strongly suggest that those genes belong to the same regulatory circuit than the *NAG* genes, although there is yet no direct proof of their involvement in NAGA metabolism.

The *Ylnag5* mutation also caused an increase in the expression of the gene *YALIF04169g*, encoding a protein similar to *S. cerevisiae* glycogen phosphorylase. Since this increase was observed in exponentially growing cultures, the behaviour is different from that seen in *S. cerevisiae*, in which the expression of the corresponding gene, *GPH1*, increases during the late exponential phase of growth [36]. Bhutada et al. [37] also reported a difference in the temporal accumulation of glycogen between *Y. lipolytica* and *S. cerevisiae*; *Y. lipolytica* accumulated glycogen during the exponential phase of growth [38], while *S. cerevisiae* did it at the entrance in the stationary phase of growth [39]. Both results indicate important differences between these yeasts in the regulation of the pathway of glycogen synthesis.

Among the overexpressed genes, a certain number of them encoded proteins related to the cell wall and of proteins containing a GPI anchor. A plausible explanation could be that altered regulation of expression in the *Ylnag5* mutant mimics a signal that in the wild type is caused by the presence of NAGA in the medium and leads to an activation of genes encoding these families of proteins functionally linked to NAGA metabolism.

An interesting result derived from the use of heterologous proteins in this work has been the unequivocal identification of the protein G4NB55 from *M. oryzae* as a NAGA kinase as our previous phylogenetically analysis predicted [19]. When expressed in a *Ylnag5* mutant, this protein behaved similarly to the enzyme from *Y. lipolytica* for both the metabolic and the regulatory function. Recent studies on the impact of the NAGA catabolic pathway in the infection of plants by *M. oryzae* revealed that defects in this pathway severely reduce virulence of this fungus but did not identify which of the hexose kinases annotated in the genome of *M. oryzae* is the actual NAGA kinase [40]. The importance of our finding is to be seen in the context of *M. oryzae* pathogenicity. This fungus is responsible for the annual loss of 10–30% of the global rice harvest [41]. Our result also shows that the generated *Ylnag5* mutant is a useful tool for correctly classifying proteins annotated in databases as “hexose kinase” or “hexokinase-like”.

The concept of moonlighting proteins [20,21] has broadened the perspectives on the functions of these molecules, showing that many proteins with a metabolic function participate in different unrelated processes [42,43,44]. The behaviour of YlNag5 indicates that it moonlights in the repression of the synthesis of the enzymes of the NAGA utilization pathway. Commichau and Stülke proposed to name as trigger enzymes those moonlighting proteins that participate in the control of gene expression, independent of their mechanism of action [44]. The NAGA kinase of *Y. lipolytica* could fit in this category.

## 4. Materials and Methods

### 4.1. Strains and Culture Conditions

The yeasts strains used during this work are shown in Table 3. The double *Ylngt1::HYG nag5::LEU2* mutant, strain CJM 2099, was obtained by a cross between the single mutants, followed by sporulation of the diploid and selection among the progeny. Strain CJM5012, a *Ylnag5* strain carrying a fusion of the promoter of the *YlNAG5* gene to the *lacZ* gene, was constructed as follows. First, we generated, in strain PO1a, a deletion of the *YlNAG5* gene using a cassette with the hygromycin resistance marker. Then the fusion of the *YlNAG5* promoter to *lacZ* was integrated at the *LEU2* locus. The resulting strain conserved the *ura3* mutation as marker for subsequent transformations. Growth medium was 0.17% yeast nitrogen base, YNB (Difco, Detroit, MI, USA) with the addition of 2% (*w*/*v*) glucose or NAGA as carbon sources, auxotrophic requirements (20 µg/mL) as required by each strain, and 2% agar in the case of solid media. Yeast transformation was done using the lithium acetate method basically as in [45]. For selection of transformants resistant to hygromycin B, 200 mg/L of the antibiotic was added to the plates.

### 4.2. Plasmid Construction

DNA manipulations were done by standard techniques. DNA corresponding to the different genes studied was obtained by PCR using adequate oligonucleotides for each case (Supplementary Table S2) and inserted in plasmid pCL49 [13]. This vector carries a *YlTEF* promoter followed by a *YlXPR2* terminator cassette and contains a *YlURA3* selection marker. The Uniprot accession numbers of the heterologous NAGA kinases used are: *Arabidopsis thaliana*, Q8LGE0; *Magnaporthe oryzae*, G4NB55; *Homo sapiens* (short), Q9UJ70; *Homo sapiens* (long), Q9UJ70-2. Note that in the case of the *A. thaliana* kinase, a polyHis tag was added at the N-terminus of the protein. The sequence encoding the human NAGA kinase was synthesized by Invitrogen GeneArt Gene Synthesis with its codon usage adapted to that of *Y. lipolytica*. All PCR products were sequenced. The different constructions were introduced in the yeast strain CJM 753.

### 4.3. Preparation of Extracts and Enzyme Assays

Cell free extracts for the measurement of enzymatic activities were prepared by vortexing the yeast with glass beads in 50 mM potassium phosphate, 1 mM EDTA, 1 mM DTT pH 7.6 in five cycles of 1 min of vortexing and 1 min in ice. When NAGA kinase activity was to be assayed, 1 mM PMSF and 0.1 mM NAGA were added. The extracts were centrifuged at 4 °C for 15 min at 13,000× *g*, and the supernatant was used for determination of enzymatic activity and for Western blot analysis (see below).

Glucosamine-6-P deaminase activity was assayed spectrophotometrically at 340 nm, coupling the production of fructose-6-P to an auxiliary system generating NADPH essentially as in [46]. NAGA kinase was assayed by an adaptation of the method described by [47] that determines residual NAGA after incubation with the enzyme preparation. The assay mixture was 250 mM phosphate pH 7.5, 4 mM NAGA, 20 mM ATP-Mg and an adequate amount of extract. A blank without ATP for each sample was carried in parallel. The mixtures were incubated at 30 °C for different times, and the reactions were stopped by the addition of ZnSO_4_ (final concentration 4.5%); the NAGA-6P formed was precipitated with 70 mM Ba(OH)_2_ and centrifuged for 5 min at 3300xg. The supernatant was used to assay residual NAGA by the procedure of [48] as modified by [49]. The Ba(OH)_2_ solution was prepared as described by [50].

Protein was assayed using the commercial BCA protein assay kit (Pierce).

### 4.4. β-Galactosidase Plate Assay

The permeabilization method of [51] was adapted for assay on plates as follows. To 7.5 mL of 1.8% melted low melting point agarose, 2 mL of 1.25% N-lauroylsarcosine sodium salt (Sigma-Aldrich, Madrid, Spain) in 0.5 M potassium phosphate pH 7.0 was slowly added with stirring at 43 °C. When homogenously mixed, 0.5 mL of 2% X-gal dissolved in dimethylformamide was added with slow stirring. The mixture was carefully poured over a plate in which colonies or patches of the corresponding strains had grown in the required medium. The plate was covered with an aluminium foil, allowed to solidify (3–5 min), incubated at 28 °C and colour development followed. Viable biomass could be recovered from the treated yeasts.

### 4.5. Antibody Production

Antibodies against YlNag5 were raised in rabbits by Davids Technologies (Regensburg, Germany) using the peptides KDQLKKTEQDQDKYIVNCEAS (1) and AEVADLETADSGELDYVQD (2) identified as potentially immunogenic. The antisera were tested against the two peptides, and then affinity was purified using a column of Sepharose bound to the peptide (1) that produced the strongest antibody response.

### 4.6. Western Blot Analysis

Extracts were obtained as described above. To prepare the samples for Western blot, 75 µg of protein from each extract were precipitated with 5% TCA, kept on ice for 15 min and then centrifuged at 20,000× *g* 15 min. The pellet was washed with cold water and resuspended in 1X Laemmli solution at 2 µg/µL of protein, heated at 42 °C for 15 min and then loaded in the gel. Preparation of gels, electrophoresis, transfer to membranes and incubations with antibodies were performed using established protocols. Separation of proteins was carried out in 10% polyacrylamide gels with SDS using 25 µg protein for each sample. After electrophoresis, they were transferred to nitrocellulose membranes (Protran 0.45 µm pore diameter, Sigma-Aldrich, Madrid, Spain) and were incubated overnight with primary antibodies anti-His tag (Aviva Systems Biology, OAEA00010, BioNova Científica, Madrid, Spain) 0.1 µg/mL, or anti-human N-acetylglucosamine kinase antibody (Novusbio NBP1-89750, BioNova Científica, Madrid, Spain) 1/10,000, or anti-YlNag5 antibody 1/15,000 in the cold, with gentle agitation.

As loading control, a polyclonal rabbit antiserum anti *Y. lipolytica* glyceraldehyde-3-P dehydrogenase (GAPDH) (a gift from A. Soukri, University Hassan II, Casablanca, Morocco) was used diluted 1/180,000. Commercial ECL Western blotting detection reagent RPN2134 was used to develop the signal.

### 4.7. Random Mutagenesis of YlNAG5

A library of random mutagenized variants of *YlNAG5* was generated using the “Error-prone mutagenesis” kit from Jena Biosciences (Jena, Germany). To the *YlNAG5* sequence a Kozak sequence was added, and the restriction sites XbaI and SnaBI were placed, respectively, at the 5’ and 3´ ends of this fragment. The sites XbaI and SnaBI were also created into plasmid pCL49 [13]. Both constructs were sequenced. The *YlNAG5* construct was used as template for the random mutagenesis reaction. The reaction, in a final volume of 25 μL, contained 6 ng of template, 20 pmol of primers and 1 unit of Taq polymerase in the error-prone buffer, as recommended by the supplier. The PCR program was as follows: 2 min at 94 °C, followed by 30 cycles of 30 s. at 94 °C, 30 s. at 61 °C and 90 s. at 72 °C. After completion of the reaction, the PCR products were cut with XbaI and SnaBI and ligated into the modified pCL49 digested with the same enzymes. The resulting plasmids were used to transform yeast strain CJM 5012.

### 4.8. RNA-Seq Experiments

Total RNA was extracted from *Y. lipolytica* cultures in exponential phase of growth in minimal medium with glucose as carbon source. The RNA was extracted using the Speedtools total RNA extraction kit (Biotools, Madrid, Spain). The integrity of the RNA was assessed using a 2100 Bioanalyser (Agilent). Only RNAs with a RIN > 7.7 were used. The cDNA library was made with the TruSeq LT kit (Illumina, San Diego, CA, USA). Given the relatively high GC content of the *Y. lipolytica* genome, the following modifications were made to the standard protocol: (1) priming time with oligodT was extended to 10 min, (2) extension with reverse transcriptase was conducted at 45 °C, and (3) the subsequent library amplification was carried out for 15 cycles. Sequencing was performed in a MiSeq equipment (Illumina) with single reads of 121 cycles. FASTQ files were generated and purged from low quality reads. Mapping was carried out with Bowtie2 software (mode sensitive, local) against the *Y. lipolytica* genomic sequence (assembly GCA_000002525.1.27) imported on 28 July 2015 from http://fungi.ensembl.org. Overall alignment rate ranged from 78% to 96% (average 89%). SAM files were analysed using the software SeqMonk v0.31.0 (Babraham Bioinformatics, Cambridge, UK), to which the gff3 annotation file was also incorporated. Reads were combined and mapped onto CDS probes extended 50 nucleotides upstream and downstream. Quantification was conducted for the opposite strand. The outputs were recovered as Annotated Probe Report, which included the coding sequences (overlapping option), and were processed in Excel format.

### 4.9. Gene Ontology (GO) Analysis

Differentially expressed genes were analysed for their known functions using GO Enrichment analysis [52] from the Gene Ontology resource [53,54]. The analysis type was the PANTHER Overrepresentation Test (Released 20210224), Annotation Version and Release Date: GO Ontology database DOI: 10.5281/zenodo.4495804 Released 1 February 2021. The test type was Fisher´s exact with no correction.

## Figures and Tables

**Table 1:** Effects of expression of heterologous NAGA kinases on the derepression of *NAG* genes.

Strain	Characteristics	Specific Activity (mU/mg Protein)	
		YlNag5	YlNag1
CJM 5000	Δnag5/pCL49(void)	No activity	198 ± 12
CJM 5010	Δnag5/pCL49*NAG5*	105 ± 3	38 ± 6
CJM 5001	Δnag5/pCL49AtNAGAK	<1	53 ± 13
CJM 3001	Δnag5/pCL49MoNAGAK	9 ± 2	<1
CJM 5002	Δnag5/pCL49HsNAGAK	18 ± 3	<1

The strains were grown in glucose and Nag5 and Nag1 activities were determined as described in Section 4. The values are mean ± SD of at least three independent cultures in each case.

**Table 2:** Gene ontology enrichment analysis performed with the genes overexpressed in the *Ylnag5* mutant vs wild type.

Biological Process	Gene	Description	*Ylnag5*/WT Ratio
*NAGA metabolism*			
	*YALI0E20163g*	*NAG2*, NAGA-6-P deacetylase	49.6
	*YALI0C01419g*	*NAG1*, Glucosamine-6-P deaminase/isomerase	25.7
	*YALI0D09801g*	*NGT1*, NAGA transporter	25.6
	*YALI0F04466g*	Similar to *GIG1* from *C. albicans* and to *HTC1* (*YPL067C*) from *S. cerevisiae*	12.5
*Glycogen catabolism*			
	*YALI0F04169g*	Similar to *S. cerevisiae YPR160w GPH1*	3.4
*Cell wall organization*			
	*YALI0D01331g*	Weakly similar to uniprot|Q12303 *S. cerevisiae YLR121c YPS3* GPI-anchored aspartyl protease 3 (yapsin 3)	6.3
	*YALI0E11517g*	Similar to *S. cerevisiae* cell wall protein *CWP1*	3.3
	*YALI0F09163g*	Weakly similar to uniprot|Q12303 *S. cerevisiae YLR121c YPS3* GPI-anchored aspartyl protease 3 (yapsin 3)	3.8
	*YALI0C08899g*	Similar to uniprot|Q9Y776 C. tropicalis secreted aspartic protease 4	3.5
*Carotene catabolic process*			
	*YALI0C18029g*	Carotenoid isomerooxygenase	5.6
*Transmembrane transport*			
	*YALI0E04510g*	Similar to uniprot|Q9URL, *C. albicans CaPTR2* peptide transport protein	4.7
*ER-nucleus signalling pathway*			
	*YALI0D15334g*	Transcription factor *HMS1* related	5.2
*RNA splicing*			
	*YALI0D26576g*	Similar to uniprot|Q04675 *S. cerevisiae YMR059w SEN15* tRNA splicing endonuclease delta subunit singleton	2.3

**Table 3:** *Y. lipolytica* strains used in this work.

Strain	Relevant Genotype	Origin
CJM246 PO1a	*leu2 ura3*	C. Gaillardin, INRA, France
CJM 753	*nag5::LEU2 ura3*	[19]
CJM 2067	*ngt1::HYG*	This work
CJM 2099	*nag5::LEU2 ngt1::HYG*	This work
CJM 5012	*nag5::HYG leu2::promNAG5-lacZ*	This work
CJM 5000	CJM 753/pCL49 ^(a)^	This work
CJM 5010	CJM 753/pCL49 *YlNAG5*	This work
CJM 5001	CJM 753/pCL49 *At*NAGAK	This work
CJM 3001	CJM 753/pCL49 *Mo*NAGAK	This work
CJM 5003	CJM 753/pCl49 *Hs*NAGAK (long)	This work
CJM 5002	CJM 753/pCl49 *Hs*NAGAK (short)	This work
CJM 753F-S	CJM 753/pCL49 *Ylnag5* F320S	This work
CJM 753D-V	CJM 753/pCL49 *Ylnag5* D214V	This work

^(a)^ Plasmid pCL49 is described in Reference [13]; *At*, *Arabidopsis thaliana*; Mo, *Magnaporthe oryzae*; *Hs*, *Homo sapiens*; NAGAK, N-acetylglucosamine kinase.

## Data Availability

RNA-seq primary data can be retrieved from the Gene Expression Omnibus (GEO) repository under series entry GSE179686.

## Supplementary Materials

The following are available online at https://www.mdpi.com/article/10.3390/ijms222313109/s1.
