# Supplementary material for: The N-Acetylglucosamine Kinase from Yarrowia lipolytica Is a Moonlighting Protein
Source: Int J Mol Sci. 2021 Dec 3;22(23):13109. doi: 10.3390/ijms222313109 (PMC8658026; doi:10.3390/ijms222313109)
Supplement: Supplementary file 1 [file ijms-22-13109-s001.zip › TABLE S1.pdf]

Table S1. Mutations found in plasmids isolated from clones obtained from the transformation with the mutational library.

Clon 1	Clon 2	Clon 3	Clon 4	Clon 5	Clon 6
H 12Q	M1K	H 12 Q	K 108 M	H 28 P	I 185 T
S 303 T	T 96P	S 134 T	D115 E	N 69 Y	K 234 E
F 320 S	I 217 V	S 159 P	N 157 D	K 288 R	V 378 M
I 397 V	T 235 S	K 280 Q	D 214 V	T 360 S	
		L 340 R	F 320 Y		

The two mutations that were selected for further studies are marked in red
